# A characterized promoter suite enables metabolic rewiring of the acetogen *Acetobacterium woodii* toward ethanol production

**DOI:** 10.1186/s13068-026-02790-2

**Published:** 2026-07-13

**Authors:** Renaud Eynard, Sephora Justine, Rémi Hocq, Stefan Pflügl

**Affiliations:** 1 Institute for Chemical, Environmental and Bioscience Engineering, Technische Universität Wien, Gumpendorfer Straße 1a, 1060 Vienna, Austria; 2 Austrian Centre of Industrial Biotechnology (acib GmbH), Vienna, Austria

**Keywords:** Acetogens, Methanol, Formate, Syngas, Fluorescent reporter, CRISPR, Metabolic engineering

## Abstract

Acetogenic bacteria are promising platforms for converting gaseous and liquid one-carbon (C1) substrates such as carbon monoxide, carbon dioxide (CO_2_), formate and methanol into fuels and chemicals, but their application is limited by sparse genetic tools and poor control over metabolic fluxes. Here, we developed a modular plasmid assembly platform and a suite of characterized native, heterologous and methanol-inducible promoters for the model acetogen *Acetobacterium woodii*, enabling rapid and tunable control of gene expression together with CRISPR-based genome editing. Using an anaerobe-compatible fluorescent reporter (pFAST), promoter strength was quantified, from medium to high expression levels. Subsequently, a minimal promoter replacement at the native *adhE* (bifunctional aldehyde-alcohol dehydrogenase) locus along with deletion of the neighboring LysR-type regulator was done employing the newly characterized promoters, demonstrating that a single promoter replacement in the genome was sufficient to shift *A. woodii* from a primarily acetogenic toward a partly ethanologenic phenotype. The engineered strain was able to produce ethanol not only from fructose but also from the C1 substrates methanol + CO_2_ and formate + syngas. Together with the promoter replacement strategy, these findings underline that ethanol formation in *A. woodii* is constrained both by energy conservation and by endogenous transcriptional control. Relieving energetic bottlenecks through the choice of energetically favorable C1 substrates and decoupling *adhE* expression from its native promoter suffices to enable ethanol production from methanol + CO_2_ and formate + syngas, directly linking the Wood–Ljungdahl pathway to ethanol formation. These results illustrate how small, targeted genome edits can significantly reshape carbon flux in acetogens and positions *A. woodii* as a viable chassis for C1-based bioproduction.

**Graphical Abstract:**

**Figure:**
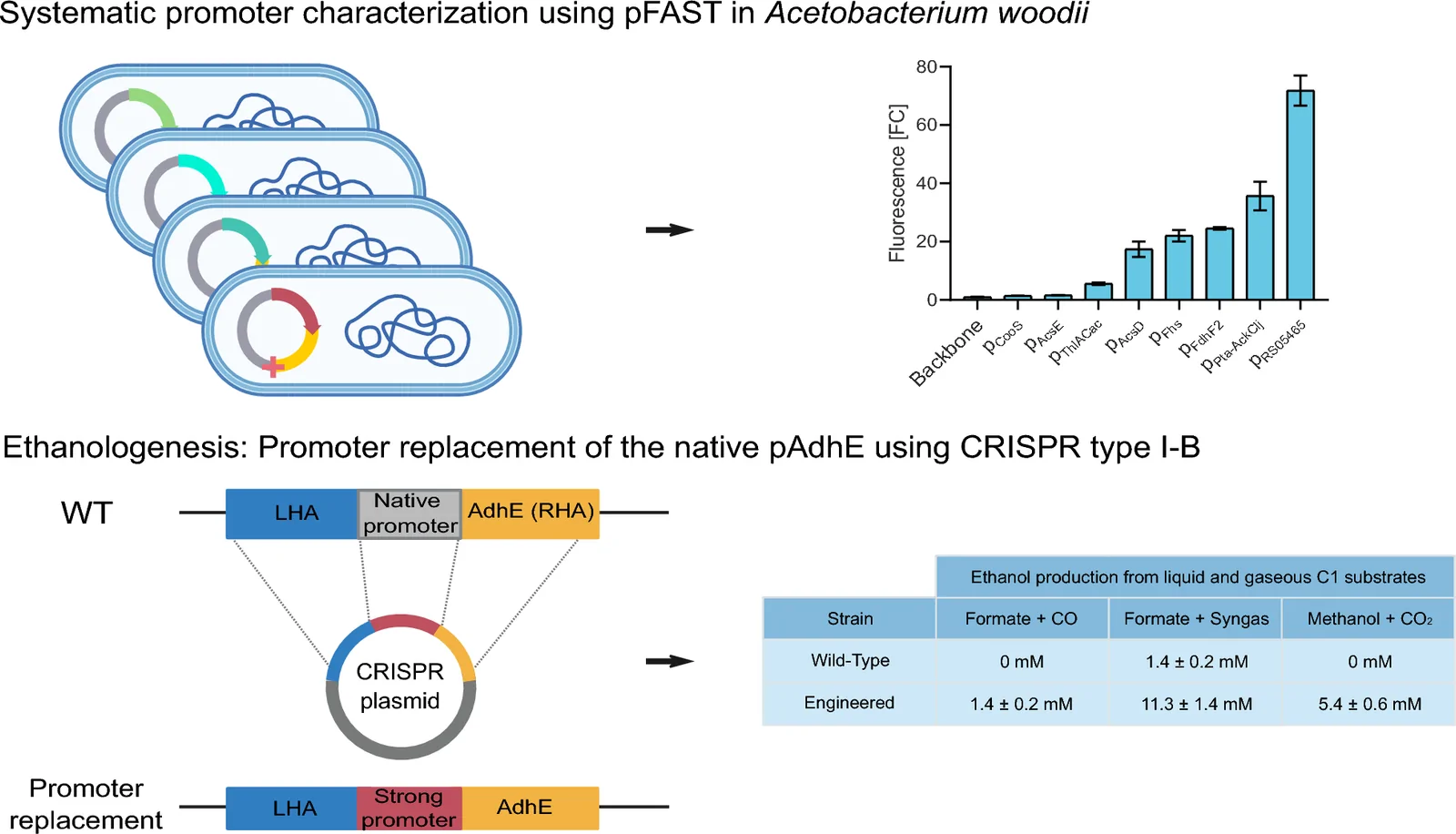

**Supplementary Information:**

The online version contains supplementary material available at https://doi.org/10.1186/s13068-026-02790-2.

## Introduction

The transition toward a circular bioeconomy has accelerated the search for robust microbial platforms that can convert gaseous and liquid one-carbon (C1) substrates into value-added chemicals and fuels [2, 56, 71]. Among these, acetogens have emerged as particularly promising biocatalysts because they channel a wide range of C1 substrates through the Wood–Ljungdahl pathway (WLP), enabling direct conversion of gases such as CO, CO_2_ and formate into acetyl-CoA derived fuels and chemicals [37, 60]. This pathway is one of the seven currently known natural carbon dioxide (CO_2_) fixation pathways [26, 64] and is widely considered the most energy-efficient, operating at the thermodynamic limit of life [51, 60, 66]. This ancient metabolic route enables acetogens to fix CO_2_ and reduce it to acetyl-CoA, a central precursor for acetate and other potential products, such as ethanol and butanol. While generally associated with gaseous substrates like H_2_ and CO from industrial flue streams [40, 44], acetogens exhibit remarkable metabolic flexibility, also utilizing liquid C1 feedstocks such as formate and methanol [59].

Among acetogens, *Acetobacterium woodii* has emerged as a model organism for studying acetogenesis and energy conservation [66]. *A. woodii* is metabolically versatile and utilizes a broad range of substrates including formate [48, 50, 52], methanol [41, 45], H_2_/CO_2_ [12], CO [8, 50], syngas [54], sugars [30] and ethanol [13] to produce primarily acetate, rendering it a potential robust platform for C1 bioconversion. The genetic toolkit for this acetogen has expanded rapidly, including robust electroporation protocols, plasmid-based expression systems and genome editing methods [4, 35, 61, 69, 70, 73]. Notably, *A. woodii* possesses a functional endogenous type I-B CRISPR–Cas system that has been repurposed for efficient, scarless genome engineering [61]. Biochemical methods and genetic tools have been used to elucidate the function of key enzyme complexes, including the essential role of the Rnf complex and the electron-bifurcating hydrogenase HydABCD in coupling the WLP to energy conservation [73, 74]. Furthermore, these tools have also been successfully used to engineer the production of industrially relevant chemicals such as acetone [1, 35], isopropanol [1, 34], lactate [47] and butyrate [20], demonstrating the significant potential of this strain as a robust biocatalyst for sustainable production of chemicals.

Some acetogens such as *Clostridium ljungdahlii* already benefit from well-established constitutive and inducible promoters (p_Tet_, p_Bgal_ or p_Choline1_) that facilitate predictable pathway expression [5, 36, 76, 77]. In contrast, despite recent progress, metabolic engineering of *A. woodii* has only begun to benefit from a systematically characterized set of promoters covering different expression levels and regulatory behaviors. Key examples include p_ThlACac_ (derived from the *Clostridium acetobutylicum* thiolase gene) and p_Pta-AckClj_, one of the few characterized promoters using a β-glucuronidase reporter activity assay [6], originating from the *C. ljungdahlii* phosphotransacetylase/acetate kinase operon. Furthermore, several inducible promoters have also been characterized using β-glucuronidase assays in the strain [6] such as the IPTG/lactose-inducible promoter p_Fac_, a lactose-inducible promoter p_BgaL_, the tetracycline-inducible promoter p_Tet_ and a lactate inducible promoter p_LctA_ [68]. This promoter palette already provides an important foundation for genetic control in *A. woodii* and has enabled the first generation of metabolic engineering studies in this organism. Building on this groundwork, the development of a broader, well-validated promoter suite with graded activities will further simplify fine-tuning of gene expression to facilitate balanced pathway fluxes, thus promoting the construction of high-performing *A. woodii* strains for sustainable biomanufacturing.

To address this gap in promoter availability for *A. woodii*, we developed, characterized and exploited a promoter suite. This suite includes native *A. woodii* promoters which were identified using previously obtained transcriptomic datasets [52], a methanol-inducible promoter (p_MeOH_1_) and heterologous promoters (p_Pta-AckClj_ and p_ThlACac_) previously used in *A. woodii* [6, 18, 20, 35]. The promoters were characterized using pFAST, a fluorescent reporter protein compatible with anaerobes [7, 15]. Following promoter characterization, we constructed ethanol-producing strains of *A. woodii* by deleting the LysR‑type regulator located immediately upstream of the bifunctional aldehyde-alcohol dehydrogenase *adhE* locus and simultaneously replacing the native *adhE* promoter with either a strong constitutive or a methanol‑inducible promoter. The ethanol production capacity of the engineered *A. woodii* strains was successfully demonstrated from sugar as well as C1 substrates.

## Materials and methods

### Bacterial strains and culture conditions

All strains used in this study are described in Additional File 1 Table S1.

*E. coli* DH10B was routinely cultivated in LB medium (10 g L^−1^ tryptone, 10 g L^−1^ NaCl, 5 g L^−1^ yeast extract) at 37 °C with carbenicillin (100 mg L^−1^), kanamycin (50 mg L^−1^), chloramphenicol (20 mg L^−1^) or erythromycin (400 mg L^−1^) as needed. For solid media, 15 g L^−1^ agar was added.

AWO_CDM, an adapted *A. woodii* strain that is routinely used as the working strain in our laboratory due to its robust and reproducible growth on chemically defined media with C1 substrates was obtained from a grown DSM 1030 culture in complex carbonate-buffered medium (CY) supplemented with fructose. Cells were sequentially transferred twice in complex phosphate-buffered medium (PY) supplemented with carbon monoxide and formate, then four times in chemically defined phosphate-buffered medium (PY) without yeast extract. The culture exhibiting the highest OD_600_ and growth rate was transformed with the backbone B007 and subsequently cured.

*A. woodii* was handled anaerobically in a vinyl anaerobic chamber (Coy lab, Grass Lake, MI, USA) or in a Concept 500 anaerobic workstation (Baker Ruskinn, Sanford, ME, USA). Strains of *A. woodii* were cultivated at 30 °C in media containing per liter: 2.0 g yeast extract, 0.5 g cysteine-HCl x H_2_O, 2 mL of trace elements, 10 mL vitamin solution (DSMZ 141) (added after sterilization), other components depending on the medium (see below) and carbon sources as indicated for each experiment. Three distinct media variants were used in this study. The complex carbonate-buffered medium (CY) contains per liter: 10.0 g NaHCO_3,_ 0.45 g K_2_HPO_4,_ 0.33 g KH_2_PO_4_, 1 g NH_4_Cl, 0.16 g MgSO_4_ × 7 H_2_O, 0.002 g CaCl_2_ × 2 H_2_O, 2 mL trace element solution and 1.0 mg FeSO_4_ × 7 H_2_O. The complex phosphate medium (PY) contains 3.5 g NaCl, 8.44 g K_2_HPO_4_, 1.76 g KH_2_PO_4_, 1 g NH_4_Cl, 0.16 g MgSO_4_ × 7 H_2_O, 0.002 g CaCl_2_ × 2 H_2_O, 2 mL trace element solution and 1.0 mg FeSO_4_ × 7 H_2_O. Na^+^-depleted complex phosphate-buffered medium (PY-Na^+^) is identical to PY except that NaCl was replaced by 12.01 g KHCO_3_. The trace elements solution (based on DSMZ 141) contained per liter: 15.0 g nitrilotriacetic acid, 5.0 g MnSO_4_ x H_2_O, 1.52 g CoCl_2_ × 6 H_2_O, 1.8 g ZnSO_4_ × 7 H_2_O, 0.1 g CuSO_4_ × 5 H_2_O, 0.1 g H_3_BO_3_, 0.1 g Na_2_MoO_4_ × 2 H_2_O, 0.3 g NiCl_2_ × 6 H_2_O, 0.0003 g Na_2_SeO_3_ × 5 H_2_O and 0.0004 g Na_2_WO_4_ × 2 H_2_O. The vitamin solution contained per liter: 2 mg biotin, 2 mg folic acid, 10 mg pyridoxine–HCl, 5 mg thiamine-HCl, 5 mg riboflavin, 5 mg nicotinic acid, 5 mg D-Ca-pantothenate, 0.1 mg vitamin B_12_, 5 mg p-aminobenzoic acid and 5 mg DL-α-lipoic acid.

Plates were made using complex phosphate medium (PY) supplemented with 15 g L^−1^ Kobe agar (Carl Roth, Karlsruhe, Germany). Thiamphenicol (20 µg mL^−1^) or erythromycin (50 µg mL^−1^) was added when required.

12-mL Hungate tubes, 125-mL serum bottles or 24-well plates were used, respectively, for promoter characterization, *A. woodii* cultivation and colony screening after transformation. Following each inoculation, unless otherwise stated, cultivated flasks were flushed and pressurized to 1 bar overpressure with a gas mixture containing 20% CO_2_ and 80% N_2_. Cultures were incubated at 30 °C on a rotary shaker at 100 rpm.

For the cultivation of *A. woodii* for ethanol production, batch fermentations were conducted in 125 mL anaerobic serum bottles containing 50 mL (20 mL for syngas and CO experiments) of the appropriate medium, with gas phases as described in the text. For all methanol cultivations, the headspace was flushed and pressurized to 1 bar with N_2_/CO_2_ (80/20), as methanol utilization in *A. woodii* has previously been shown to be strictly dependent on the presence of CO_2_ [41]. Cultures were incubated for up to 13 days. At each sampling point, growth was monitored by measuring the optical density at 600 nm (OD_600_) and HPLC was used to quantify the different substrates consumption (except gases) and the production of metabolites to evaluate metabolic flux redirection. Overpressure was monitored with a manometer.

For strains involving characterization of promoters, OD_600_ and fluorescence were measured to quantify induction levels. Samples were additionally harvested at all time points to monitor substrate consumption during the methanol-inducible promoter characterization (fructose, formate, ethanol and methanol).

### In silico identification of *A. woodii* promoters

Native promoters of *A. woodii* were selected and designed based on genome-scale transcriptomic datasets for this strain [52, 67]. In particular, transcription start site (TSS) positions were taken from the TSS mapping reported by [67], which we used to delineate the promoter regions analyzed in this study. Native promoters were selected to cover a wide range of expression, from medium to high expression level on fructose (between ~700 TPM and ~9000 TPM).

After identifying the TSSs, the primers used to amplify the different promoters were designed to amplify the upstream region of each gene including the TSS, the -35 and -10 recognition signal and an additional 50 bp upstream of the -35 signal. Identification of the -35 and -10 signals was done using the σ^70^ consensus sequence established in *Clostridia* [33] and performed in silico using CLC Workbench (Qiagen, Hilden, Germany).

Based on the information related to the methanol operon of *A. woodii* [41], we identified several -35 and -10 signals for the promoter regulating this operon. Therefore, the entire sequence (430 bp) upstream of the first gene of the methanol operon (Awo_c22820) until the end of the CDS (Awo_c22810) upstream of the gene and the entire sequence (93 bp) localized between two CDS (Awo_c22780, Awo_c22770) were amplified by PCR and cloned in a level 1 plasmid. Throughout this study, these two promoter regions are referred to as p_MeOH_1_ and p_MeOH_2_, respectively.

### Plasmid construction with Golden Gate assembly

Plasmids and DNA parts used in this study are described in Additional File 1 Table S1. A list of all the primers used and their sequences are available in Additional File 1 Table S2. All plasmids were constructed using Golden Gate assembly.

Each part was amplified by conducting PCRs on the A. woodii genome (B7 High Fidelity polymerase, Biozym, Hessisch Oldendorf, Germany) or synthesized (Twist Biosciences, San Francisco, CA, USA) and cloned in a backbone plasmid with a compatible restriction enzyme (BbsI/BsaI/Esp3I) and appropriate fusion sites (Additional File 1 Table S3). Promoters, coding sequences and terminators were cloned individually in level 1 backbone (BB1) using BsaI assembly using unique fusion sites 1–2, 2–3, and 3–4, respectively.

For the assembly of the plasmids used for the promoters characterization, different transcription units expressing an *A. woodii* codon-optimized pFAST gene [7] were assembled with the different promoters and a BBa_B1001 synthetic terminator using a BbsI assembly into a level 2 backbone plasmid (BB2_A_1_4_B). Finally, these cassettes were cloned in a level 3 backbone plasmid (B007) with the help of two connectors (BB2_BC and BB2_CD) using a BsaI assembly to assemble an *A. woodii* expression plasmid. The *E. coli*–*A. woodii* shuttle vector B007 was obtained by modifying pMTL83241 [29] through insertion of an annealed primer pair into the BamHI and NcoI sites in order to add two BsaI recognition site with the recipient sites AD, thereby maintaining the lacZ reading frame and enabling blue/white screening.

For the knock-in and knock-out experiments conducted with the endogenous Type I-B CRISPR/Cas system of *A. woodii*, CRISPR plasmids were assembled according to previously established guidelines [61]. PAM sequences (35 bp downstream of a CCA motif) for the different targets were identified using CLC Workbench (Qiagen, Hilden, Germany). Spacer sequences were generated by annealing two primers carrying the first and last four nucleotides (AAAT and ATTT) of *A. woodii* endogenous CRISPSR system type I-B repeat units on their overhangs, resulting in sequences identical to the selected protospacers. These spacers were inserted into a vector expressing the *A. woodii* CRISPR/Cas system using an Esp3I assembly, which contains the *A. woodii* CRISPR leader sequence and direct repeat region required for crRNA generation. The resulting plasmids were designated BB2_leader1 to BB2_leader6. Final CRISPR plasmids were assembled by BsaI Golden Gate assembly using the BB2_leader plasmids (linker A–B), the repair DNA template consisting of a left homology arm (PCR fragment, linker B–C) and a right homology arm (PCR fragment, linker C–D for knock-out and linker D–E for knock-in) and where applicable, the knock-in fragment (PCR fragment, linker C–D) positioned between the homology arms. For the *adhE* promoter replacement and *lysR* knock-out, PCR fragments were used for assembly because a BsaI site was present within the right homology arm. This site was removed by introducing a single base substitution through custom primer overhangs (site X), while preserving the native amino acid sequence. The use of PCR fragments also eliminated an additional cloning step, enabling faster construction of the level 3 plasmids. The *E. coli*–*A. woodii* shuttle vector used for the level 3 plasmids assembly, B040 (linker A–D) and B041 (linker A–E), were constructed by combining several parts by BbsI Golden Gate assembly: a BsaI-compatible A–D/A–E linker [65], the pCD6 Gram-positive replicon from pMTL84422 [29], the thermostable chloramphenicol resistance marker catP (synthesized) and a ColE1 Gram-negative origin of replication [65].

### *A. woodii* transformation

Fifty mL of competent cells were prepared for each transformation with a culture of *A. woodii* with an OD_600_ between 0.2 and 0.4 (ONDA V-10 Plus, Giorgio Bormac, Carpi, Italy). After rapidly cooling the culture on ice, the cells were centrifuged at 4 °C at 5000*g* for 8 min. Supernatant was discarded, cell pellets were washed with 50 mL of ice-cold SMP buffer (pH 5.8, 270 mM sucrose, 1 mM sodium phosphate, 1 mM MgCl_2_ × 6 H_2_O) and centrifuged again in the same conditions. After discarding the supernatant, the cell pellet was resuspended in ice-cold SMP buffer at a volume of 200 µL per planned electroporation (i.e., 200 µL × number of transformations for each strain transformed). Aliquots of 200 µL of these competent cells were mixed with 4 µg plasmid DNA in 2-mm cuvettes, the cells were shocked (1.0 kV, 200 Ω, 50 µF) using a Bio-Rad Gene Pulser Xcell electroporator (Bio-Rad, Hercules, CA, USA) and were instantly transferred to 10 mL of complex carbonate medium without carbon source or antibiotic in 12-mL glass Hungate tubes. Hungate tubes were flushed and pressurized at 1 bar with 80% N_2_ and 20% CO_2_ and incubated at 30 °C on a rotary shaker at 100 rpm. After 6 h of regeneration, the cells were centrifuged at room temperature (5000*g*, 2 min) and resuspended in 100 µL of complex carbonate medium. 20 µL and 50 µL of cells were then spread on agar plates with the corresponding antibiotic. These plates were then incubated at 30 °C in a 2.5 L anaerobic jar (Oxoid, Hampshire, UK).

Colonies appeared on the plates usually after five (erythromycin/pCB102) or 12 (Thiamphenicol/pCD6) days. These colonies are first screened by re-inoculating them in 1 mL complex carbonate medium without antibiotic for the CRISPR plasmids or with erythromycin for the pFAST overexpression plasmids and were supplemented with fructose and vitamins in a 24-well plate (CytoOne, CC7672-7524, Starlab International GmbH, Hamburg, Germany). Well-plates were incubated in a custom-made anaerobic metal jar pressurized to 1 bar overpressure with 80% N_2_ and 20% CO_2_ and incubated at 30 °C. After two days, PCR was performed on cell lysates to verify plasmid presence and to screen for the desired genomic deletion or insertion. 1 µL of cell lysate was used as PCR template. Cell lysates were prepared by mixing 100 µL of cells with the same volume of 10 mM TE buffer (pH 8) and boiled at 100 °C for 20 min. Upon successful validation, 10 µL of culture were streaked another time on agar plates and incubated at 30 °C to cure the cells of residual plasmids. After colony formation, the strains were genotyped again and then validated by Sanger sequencing (Microsynth AG, Balgach, Switzerland) and stored in 10% DMSO (v/v) at − 80 °C.

### Analytical methods

Samples for HPLC were centrifuged (16,000*g*, 15 min, 4 °C) to remove biomass. The supernatant was acidified with 40 mM H_2_SO_4_ at a 9:1 ratio to match the mobile phase conditions for accurate and reproducible quantification. Metabolites were separated using an Aminex HPX-87H column (300 × 7.8 mm; Bio-Rad, Hercules, CA, USA) maintained at 60 °C, with an isocratic flow of 0.6 mL min^−1^ using 4 mM H_2_SO_4_ as the mobile phase. Compounds were detected using a Refractomax 520 refractive index detector (Thermo Fisher Scientific, Waltham, MA, USA) mounted on a Vanquish Core Quaternary HPLC system. Analyte concentrations were quantified using external standards (fructose, formate, methanol, acetate, ethanol, pyruvate, and lactate) and data were processed using Chromeleon software (version 7.3.2, Thermo Fisher Scientific, Waltham, MA, USA).

Fluorescence of pFAST was measured as described previously [31]. Briefly, 5 OD units of cells were harvested in late exponential phase (starting from OD_600_ of 0.8), unless otherwise stated. The cells were centrifuged at room temperature (5000*g*, 2 min), the supernatant was discarded and each pellet were resuspended in 100 µL 70 mM Na^+^ phosphate pH 7.4 and 5 μΜ HMBR (hydroxymethyl bilane-related bilin chromophore) (Twinkle Factory, Paris, France) and placed in a 96-well plate (ref: 655209, Greiner, Kremsmünster, Austria). Fluorescence measurements were conducted with Tecan (Infinite M200, Tecan, Männedorf, Switzerland) as described before. Results were normalized using the fluorescence value obtained from cells harboring the empty BB3 backbone used for the pFAST plasmids assembly.

Carbon and electron balances were calculated using the number of carbon atoms and the available electrons for substrates and products: fructose (C_6_H_12_O_6_, 6 carbon atoms, 24 available electrons), methanol (CH_3_OH, 1 carbon atom, 6 available electrons), formate (CHOO^−^, 1 carbon atom, 2 available electrons), acetate (C_2_H_4_O_2_, 2 carbon atoms, 8 available electrons), ethanol (C_2_H_6_O, 2 carbon atoms, 12 available electrons), pyruvate (C_3_H_4_O_3_, 3 carbon atoms, 10 available electrons), lactate (C_3_H_6_O_3_, 3 carbon atoms, 12 available electrons) and carbon dioxide (CO_2_, 1 carbon atom, 0 available electrons). Biomass formation was included in the carbon and electron balances by converting OD_600_ values into biomass concentrations using a previously established correlation of 0.38 g dry cell weight per liter per unit of OD_600_ [52], an assumed carbon content of 45% (w/w) for *A. woodii* biomass [27] and a degree of reduction of 4.15 mol electrons per mol of biomass carbon [62]. CO_2_ production was taken into account using the expected stoichiometry for product formation. In detail, when fructose served as substrate equimolar formation of CO_2_ was assumed for each mol of acetate or ethanol formed. For methanol + CO_2_, 2/3 mol CO_2_ was considered per mol acetate formed and no CO_2_ production for ethanol. For formate, 2 mol CO_2_ was assumed per formate consumed. Yeast extract was neglected in the calculation of the carbon and electron balances. Carbon balances were calculated as: Carbon balance [%] = ((∑_i​_(C_i​*_n_i_​)) + biomass)/(C_substrate​*_n_substrate,consumed_​) × 100, where C_i_​ is the number of carbon atoms in product, C_i_​⋅n_i_ is the number of moles of product i, C_substrate_ is the number of carbon atoms in the organic substrate and n_substrate,consumed_ is the number of moles of substrate consumed.

Electron balances were reported as the ratio of total electrons recovered in products (reduced and oxidized) to electrons supplied by the consumed substrate. Electron balances were calculated as: Electron balance [%] = ​((∑_i​_(v_i_​_​*_n_i_​)) + biomass)/(v_substrate_​_​*_n_substrate,consumed_​)*100, where v_i_ is the number of available electrons per mole of product I (from its degree of reduction), n_i_ is the number of moles of product i, v_substrate_ is the number of available electrons per mole of substrate and n_substrate,consumed_ is the number of moles of substrate consumed.

## Results

### A modular Golden Gate assembly system for *A. woodii*

**Fig. 1:**
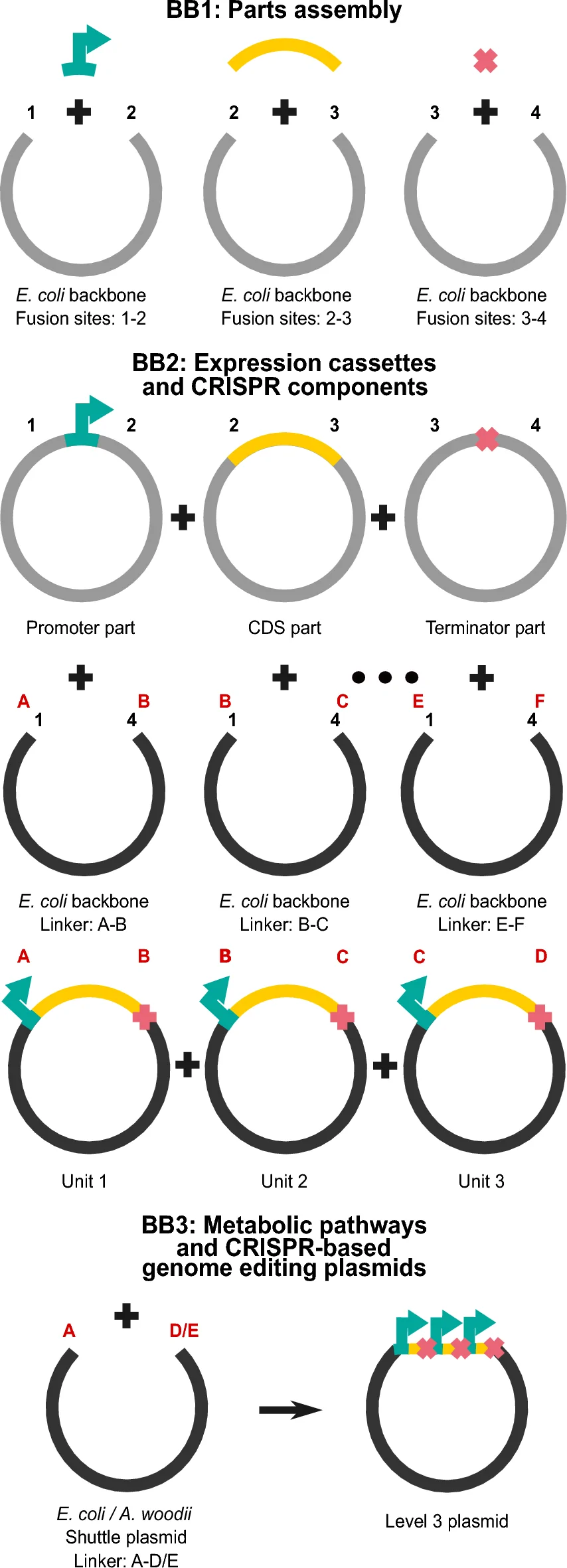
Scheme of the multi-level Golden Gate system for assembly of *A.* *woodii.* Metabolic pathways expression plasmids or CRISPR-based genome editing plasmids. Level 1: individual genetic parts, including promoters (green), coding sequences (yellow), terminators (red), are assembled into vectors using fusion sites (e.g., 1-2, 2-3, 3-4) (BB1). Level 2: assembly of functional expression cassettes, combining promoter, coding sequence (CDS) and terminator, or CRISPR components into vectors (BB2) containing specific linker sequences (from A–B to E–F) for a modular final assembly of up to five units. Level 3: multiple expression cassettes, or CRISPR components are assembled into final *E. coli*/*A. woodii* shuttle plasmids (BB3) for pathway overexpression or CRISPR-based genome editing. Details on plasmid components and overhang sequences can be found in Additional File 1 Table S1 and Table S3, respectively

Golden Gate cloning enables efficient and modular assembly of multiple DNA fragments by exploiting Types IIS enzymes and T4 DNA ligase in a single reaction [23]. For this reason, we developed a plasmid library designed and based on the GoldenMOCS (Golden Gate derived Multiple Organism Cloning System) [65]. This system consists of three assembly levels (level 1 = BB1, level 2 = BB2, level 3 = BB3) (Fig. 1) and uses the type IIS enzymes BsaI (for assembly of BB1 and BB3 plasmids) and BbsI (for BB2 plasmids). BB1 plasmids are entry vectors for individual genetic parts (promoters, coding sequences, terminators) with unique BsaI restriction sites. Assembly into BB2 vectors yields functional expression cassettes or may be used to clone different CRISPR components (e.g., homology arms, gRNA expression cassettes). The final BB3 vectors integrate multiple expression cassettes into final *E. coli/A. woodii* shuttle plasmids, enabling the generation of complex constructs such as metabolic pathways for overexpression or CRISPR-based genome editing plasmids. To integrate the established endogenous Type I-B CRISPR/Cas system of *A. woodii* [61] in our cloning workflow, we generated a BB2 plasmid containing the CRISPR leader sequence followed by two repeat units separated by two Esp3I recognition sites, allowing for efficient spacer insertion to assemble a functional CRISPR array.

For transformation of *A. woodii*, two different BB3 plasmids were developed by adapting the pMTL80000 modular shuttle vectors [29], each tailored for distinct applications. For gene overexpression, we generated a BB3 suite with the *ermB* erythromycin resistance gene [35] and the pCB102 Gram-positive replication origin, which is highly stable in *A. woodii* [4]. For genome-editing applications, a second vector set was constructed carrying a thiamphenicol resistance marker *catP* [19] and the unstable pCD6 origin [4]. Unstable origins of replications can be exploited in systems requiring transient replication, such as CRISPR-mediated genome editing, as plasmid curing is typically required after each round of modification.

### Expanding *A. woodii* promoter toolbox

**Fig. 2:**
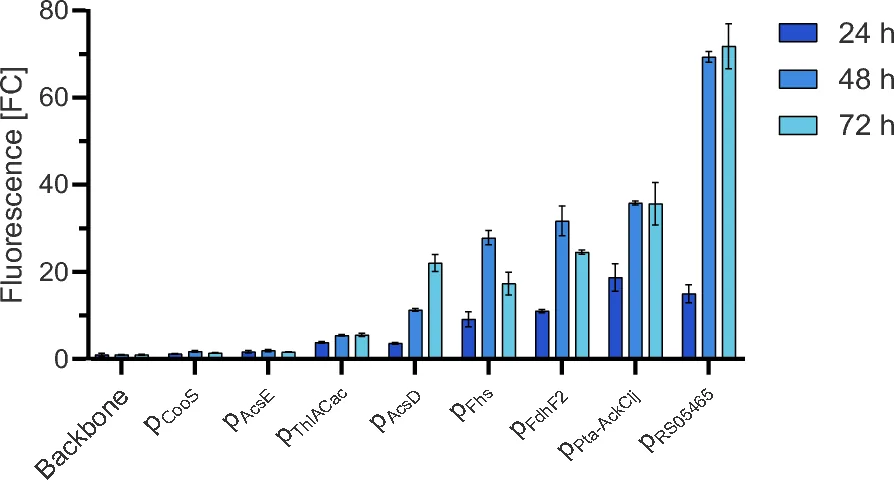
Characterization of promoters in *A. woodii*. Fluorescence of a library of pFAST expression cassettes with different promoters was measured with a plate reader. Fluorescence is shown as the fold change of the average of biological triplicates over the average fluorescence measured for an *A.* *woodii* population bearing only the empty backbone B007 used to construct the pFAST plasmids. Cells were grown in 10 mL complex carbonate medium (CY), supplemented with 25 mM of fructose and the headspace was flushed with N_2_. Cells were incubated at 100 rpm and 30 °C for 72 h. Data represent average ± standard deviation of three biological replicates. Corresponding growth profiles of these cultures and fluorescence signal raw data can be found in Additional File 1 Table S6

To expand the promoter toolbox for *A. woodii*, we characterized six native promoters and two heterologous promoters (p_Pta-AckClj_ from *C. ljungdahlii* and p_ThlACac_ from *C. acetobutylicum*) [6, 20, 35], using our hierarchical Golden Gate assembly system to overexpress pFAST (Fig. 2). The native promoters (p_CooS_, p_AcsE_, p_AcsD_, p_Fhs_, p_FdhF2_ and p_RS05465_; respective locus tag: Awo_c10740, Awo_c10730, Awo_c10710, Awo_c09260, Awo_c08210 and Awo_c10770) were selected based on RNA-seq data [52] and transcription start site (TSS) analysis [67]. This selection was made to cover a broad range of expression levels, from medium to high expression levels (expressed in transcripts per million, TPM) on fructose (~ 700 TPM/p_FdhF2_ to ~ 9,000 TPM/p_AcsE_), formate (~ 1000 TPM /p_FdhF2_ to ~ 20,000 TPM /p_AcsE_) and syngas (~ 600 TPM /p_FdhF2_ to ~ 30,000 TPM /p_AcsE_). These promoters (sequences are listed in Additional File 1 Table S4) were then cloned into BB1 vectors and subsequently assembled in BB2 plasmids with the pFAST coding sequence and the BBa_B1001 synthetic terminator (iGEM), frequently used for metabolic engineering of *E. coli* [24]. These cassettes were assembled in a BB3 *E. coli*/*A. woodii* shuttle plasmid. Subsequently, these shuttle plasmids were used to transform an *A. woodii* strain adapted to grow robustly on C1 substrates in chemically defined medium (referred to hereafter as AWO_CDM). Upon successful strain generation, strains carrying all promoters from the suite described above were grown on fructose and fluorescence was measured in triplicates at three different time points (24, 48 and 72 h).

Out of the eight promoters tested, six promoters (p_Fhs_, p_FdhF2_, p_AcsD_, p_RS05465_, p_Pta-AckClj_ and p_ThlACac_) showed fluorescence signals with different strengths while the remaining two (p_AcsE_ and p_CooS_) did not produce detectable fluorescence signals beyond the background level of the empty vector control. Generally, fluorescence signals appeared after 24 h and reached their maximum at 48 or 72 h. The highest fluorescence was recorded for the promoter p_RS05465_ (Awo_c10770), expressing a putative zinc transporter in the same operon as the monofunctional carbon monoxide dehydrogenase gene (*cooS*) of the WLP, with an average 71-fold increase in fluorescence relative to the empty vector control at 72 h. The next highest fluorescence signals were observed for p_Pta-AckClj_, p_Fhs_ and p_FdhF2_ with fold changes of 36, 28 and 32, respectively. p_AcsD_ drove a moderate response with a fold change of 28 and p_ThlACac_ emitted the smallest fluorescence with an average fold change of 5. These variations in fluorescence were corroborated by transcriptomics data from fructose-grown *A. woodii* [52] which showed similar expression variations, except for p_AcsD_. Specifically, the high fluorescence intensity observed for p_RS05465_ aligns with the significant upregulation of the Awo_c10770 gene (~ 1,500 TPM) compared to Awo_c09260 (p_Fhs_, ~ 760 TPM) and Awo_c08210 (p_FdhF2_, ~ 760 TPM) during growth on fructose, which ranks among the most highly expressed gene clusters. The differences in fluorescence signal strength between p_RS05465_ and p_Fhs_, p_FdhF2_ reflect the relative mRNA abundance of their respective genes [52], suggesting that the reporter system accurately captures the transcriptional output of the different promoters. These correlations can be found in Additional File 1 Table S5.

### A methanol-inducible promoter for *A. woodii*

Reliable inducible promoter systems for *A. woodii* are desirable for applications such as controlling the expression levels of individual pathway genes. The methanol-dependent operon in *A. woodii* (locus tag: Awo_c22810 to Awo_22740), which encodes the methanol-specific methyltransferase system and is strongly induced during growth on methanol, has previously been described [41], making it a prime candidate for characterizing an inducible promoter system responsive to a highly relevant carbon and energy source. Kremp et al. demonstrated the sub-operonic structure of the methanol operon *mta*, with two putative TSSs within this locus: one (p_MeOH_1_) located upstream of *mtaR* (Awo_c22810), a gene encoding a protein containing a PocR-type ligand-binding domain-containing protein, belonging to the AraC/PocR family of transcriptional regulators [11, 17, 19] and one upstream of Awo_c22770 (p_MeOH_2_), which encodes a predicted B_12_-binding protein. Transcriptional data suggested differing strengths for these sites [41], ~3000 TPM for p_MeOH_1_ and 20,000 TPM for p_MeOH_2_. Therefore, we cloned both promoter regions into a pFAST plasmid to validate their activity. Reporter assays revealed that p_MeOH_1_ exhibited measurable fluorescence upon induction, whereas p_MeOH_2_ failed to drive detectable expression.

**Fig. 3:**
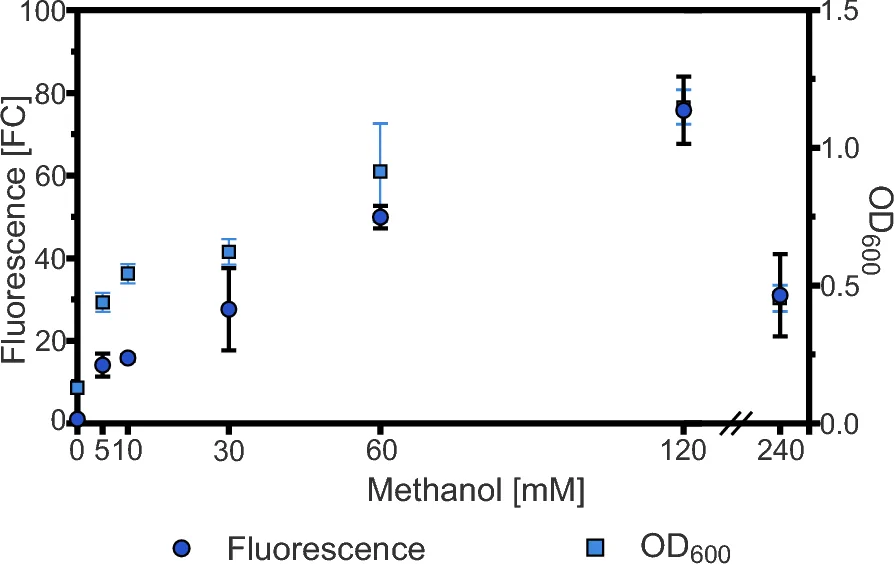
Fluorescence response of the methanol-inducible promoter (p_MeOH_1_) across a methanol concentration range measured with the pFAST reporter. Fluorescence of pFAST expression cassette driven by the methanol-inducible promoter p_MeOH_1_ was measured with a plate reader. OD_600_ was measured at each time points. Fluorescence is shown as the fold change of the average of biological triplicates over the average fluorescence measured for a strain bearing only the empty backbone B007 used to construct the pFAST plasmids across a methanol range (0, 5, 10, 30, 60, 120 and 240 mM). Cells were grown in 5 mL complex carbonate medium (CY), supplemented with methanol and the headspace was flushed and pressurized to 1 bar overpressure with N_2_/CO_2_ (80/20). Cells were incubated at 100 rpm and 30 °C for 144 h (late stationary phase). Data represent average ± standard deviation of 3 biological replicates. Corresponding growth profiles of these cultures can be found in Additional File 1 Table S7

We subsequently assessed the effect of inducer concentration on p_MeOH_1_ activity by cultivating the p_MeOH_1_-pFAST strain in medium containing increasing methanol concentrations (0, 5, 10, 30, 60, 120 and 240 mM) (Fig. 3). Fluorescence intensity increased with increasing methanol concentration up to 120 mM, from 0 to an average fold change of 76. For 240 mM, the fluorescence signal sharply declined along with the OD_600_, suggesting toxicity at higher methanol concentrations. While 120 mM methanol yielded higher fluorescence (fold change 75.8 ± 6.7) and cell densities (OD_600_ 1.15 ± 0.05) compared to 60 mM (fold change 50.0 ± 2.2; OD_600_ 0.91 ± 0.14), we selected 60 mM as the working concentration for subsequent experiments with methanol to avoid any possible growth inhibition.

**Fig. 4:**
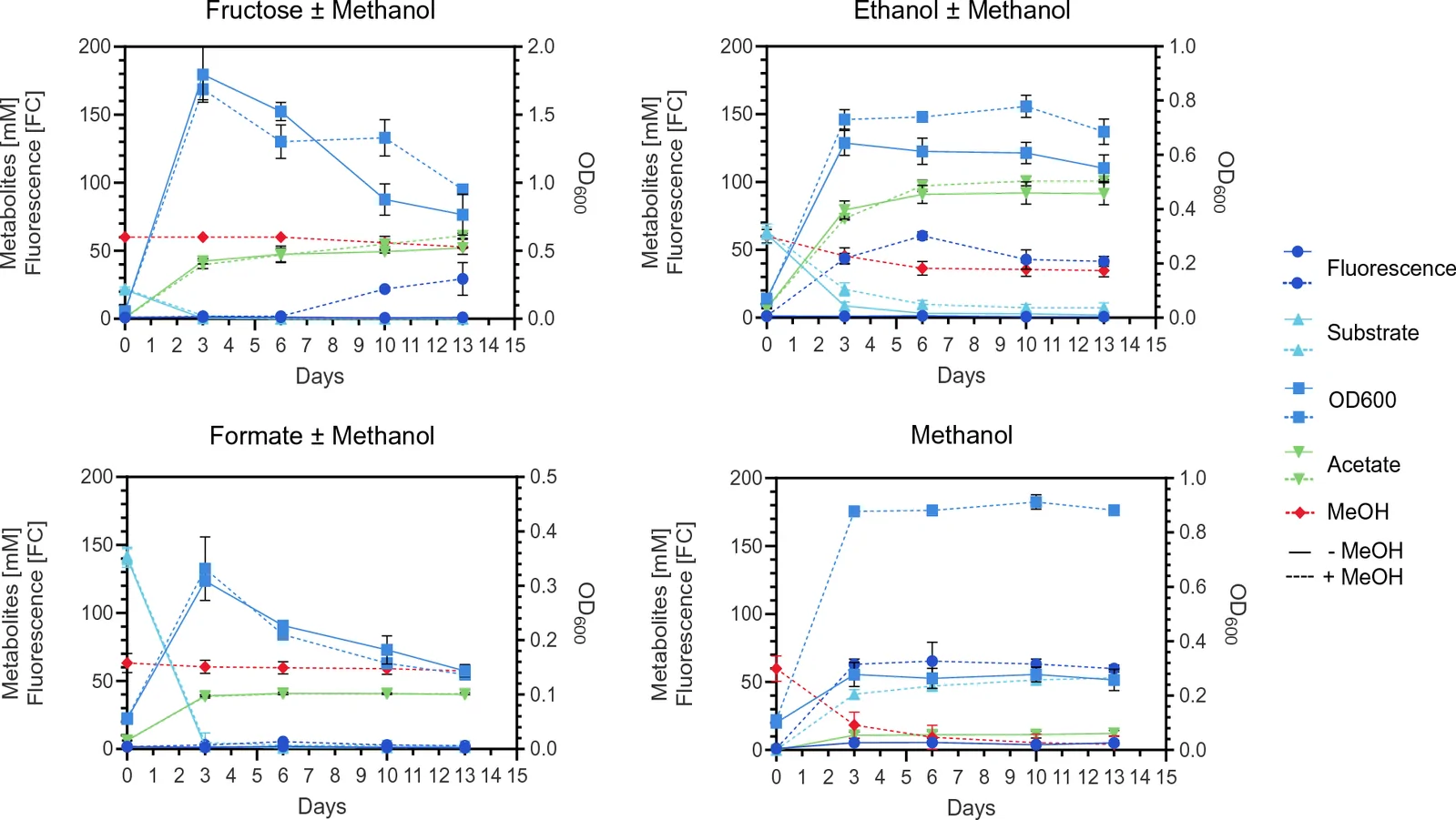
Growth, substrate consumption and pFAST activity controlled by p_MeOH_1_ of *A. woodii* in mixed-feed systems. Strains were cultivated in 50 mL in complex carbonate medium (CY) for 13 days at 30 °C, with the headspace pressurized to 1 bar overpressure with N_2_/CO_2_ (80/20). Fluorescence (●), OD_600_ (■) and substrate consumption: methanol (♦, 59.9 ± 7.7 mM) and fructose, ethanol or formate (▲, 21.5 ± 0.3 mM, 63.5 ± 2.7 mM, 142 ± 5.4 mM, respectively) were monitored in single substrate (—) and dual substrate growth (––). The fluorescence signal was normalized using a strain overexpressing the backbone used to construct the different pFAST plasmids and was cultivated in identical conditions. Data are average ± SD from biological triplicates

*A. woodii* is known for its metabolic versatility and ability to co-utilize various substrates [52]. However, little is known about the ability of *A. woodii* to co-utilize methanol together with other substrates. To evaluate the specificity and dose-dependent activation of p_MeOH_1_ and to determine if methanol acts as an inducer in the presence of alternative carbon sources, we performed a substrate co-utilization analysis (Fig. 4). We grew p_MeOH_1_-pFAST with methanol and either fructose, formate or ethanol to evaluate substrate co-consumption and the fluorescence produced by the expression of the methanol-inducible reporter in the presence of other carbon sources.

Growth was observed for all conditions tested but with distinct utilization patterns. Methanol was not consumed until complete depletion of fructose and formate, whereas it was co-consumed when ethanol was used as additional substrate. This consumption pattern is also reflected in the fluorescence signals observed. On methanol only, fluorescence was observed without delay and reached a fold change of around 63 after 3 days of cultivation, whereas only marginal fluorescence was observed for fructose, formate and ethanol in the absence of methanol. In contrast, when methanol was additionally provided, fluorescence immediately appeared when cells were co-fed with ethanol, reaching a maximum average fold change of 60 after 6 days of cultivation. In case of formate, only a negligible amount of methanol was consumed (5.4 ± 1.6 mM), leading to low fluorescence with a maximum average fold change of 5 after 6 days of incubation. For fructose + methanol grown cells, fluorescence was only observed after complete depletion of fructose and reached an average fold change of 29 after 13 days.

Together, these results demonstrate that the methanol-inducible promoter is specifically activated by methanol, with the magnitude of induction depending on the presence of certain co-substrates. While the presence of ethanol as a co-substrate allows induction, the co-substrates fructose and formate suppressed expression of pFAST. The induction was dose-dependent with respect to methanol, with maximal promoter activity at 120 mM methanol (Fig. 3). Furthermore, the analysis showed metabolic repression in *A. woodii*. Methanol was used by the cells only after fructose was fully consumed, co-consumed when ethanol was the second substrate and was not significantly consumed when co-fed with formate.

### Construction of ethanol-producing *A. woodii* strains

**Fig. 5:**
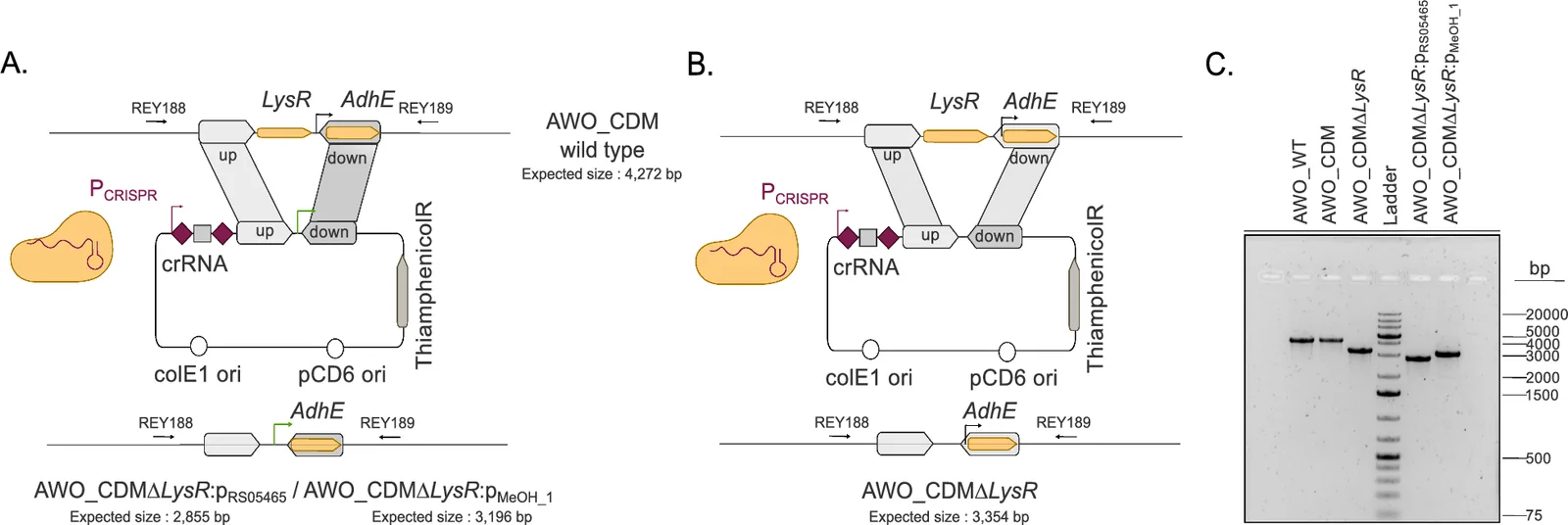
Design of the CRISPR plasmids recruiting the *A. woodii* endogenous Type I-B CRISPR/Cas system for replacing the native *A. woodii adhE* promoter. Three *A.* *woodii* strains were generated to enable ethanol production. Homology arms (up/down) were designed to either **A** insert a strong constitutive promoter (p_RS05465_) or a methanol-inducible promoter (p_MeOH_1_) and simultaneously delete the *lysR* sequence upstream of the *adhE* gene and the native *adhE* promoter, resulting in AWO∆*lysR*:p_RS05465_ and AWO∆*lysR*:p_MeOH_1_, respectively or **B** to delete only the *lysR* sequence upstream of the *adhE* gene as a control (AWO∆*lysR*). **C**. PCRs conducted on the different strains to validate the different genetic modifications

The genome of *A. woodii* contains eleven alcohol dehydrogenases, including a gene coding for the bifunctional aldehyde and alcohol dehydrogenase AdhE (Awo_c06310), which is critical for growth of *A. woodii* on ethanol [10] but is involved in ethanol synthesis in other acetogens and bacteria in general [9, 25, 39, 58]. However, expression of *adhE* is typically low when *A. woodii* grows on C1 substrates such as formate (~22 TPM) or H_2_/CO_2_ (~2 TPM) but also on sugars such as fructose (~100 TPM) because acetate is the dominant metabolic product under these conditions [45, 49, 52]. Leveraging the newly characterized promoters, we used the CRISPR-based genome editing method from Poulalier-Delavelle et al. to precisely replace the native promoter of the *adhE* gene with a strong constitutive (p_RS05465_) and an inducible promoter (p_MeOH_1_) to construct ethanol-producing *A. woodii* strains (Fig. 5). On the genome of *A. woodii*, there is a gene coding for a LysR-type transcriptional regulator (LTTR), a widespread family of bacterial transcription factors involved in metabolism and stress response [22], ending close to *adhE* (separated by only 499 bp). As this regulator might potentially be involved in the regulation of *adhE* expression, we knocked out its coding sequence in all the ethanol-producing strains.

To test ethanol production, we employed a low-carbonate, Na^+^-depleted complex phosphate medium (PY-Na^+^) previously demonstrated to suppress WLP activity and enable ethanol production in wild-type *A. woodii* [49]. Therefore, the two engineered strains, AWO_CDM∆*lysR*:p_RS05465_ and AWO_CDM∆*lysR*:p_MeOH_1,_ along with DSM 1030 (AWO_WT), our working strain (AWO_CDM) and a control deletion strain (AWO_CDM∆*lysR*) were cultivated on fructose as the substrate to assess growth, substrate consumption and metabolite formation (Table 1).

**Table 1:** Ethanol production of *Acetobacterium woodii* strains from fructose under carbonate- and Na^+^-free conditions

Strain	Fructose [mM]			Products [mM]					Yields			Balance [%]	
	Supplemented	Consumed	Biomass (X) [g L^−1^]	Acetate	Ethanol	Pyruvate	Lactate	CO_2_*	Y_Ace/Fru_ [mol mol^−1^]	Y_EtOH/Fru_ [mol mol^−1^]	Y_X/Fru_ [g mol^−1^]	Carbon	Electron
AWO_WT	62.4 ± 2.8	53.2 ± 1.2	0.27 ± 0.04	90.9 ± 2.4	4.8 ± 0.4	2.4 ± 0.1	2.0 ± 0.1	95.7 ± 2.6	1.71 ± 0.07	0.09 ± 0.01	5.16 ± 0.88	97.2 ± 4.1	68.4 ± 3.0
AWO_CDM	59.1 ± 2.6	42.3 ± 1.3	0.57 ± 0.01	53.9 ± 1.3	10.8 ± 0.6	2.3 ± 0.5	1.9 ± 0.2	64.7 ± 1.0	1.27 ± 0.06	0.26 ± 0.02	13.41 ± 0.61	89.3 ± 3.6	67.9 ± 2.8
AWO_CDM∆*lysR*	57.4 ± 0.5	40.9 ± 0.4	0.56 ± 0.01	48.3 ± 0.7	9.6 ± 0.4	3.0 ± 0.3	1.1 ± 0.8	57.9 ± 0.5	1.18 ± 0.03	0.23 ± 0.01	13.70 ± 0.26	84.0 ± 0.7	63.9 ± 0.4
AWO_CDM∆*lysR*:p_RS05465_	64.6 ± 2.8	64.6 ± 2.8	0.48 ± 0.01	28.2 ± 1.7	56.5 ± 2.1	0.7 ± 0.3	3.3 ± 0.4	84.7 ± 0.5	0.44 ± 0.05	0.87 ± 0.01	7.39 ± 0.45	86.5 ± 2.9	80.8 ± 2.3
AWO_CDM∆*lysR*:p_MeOH_1_	54.1 ± 1.4	46.5 ± 1.2	0.63 ± 0.01	62.5 ± 4.6	16.1 ± 2.0	0.7 ± 0.3	3.3 ± 0.4	78.7 ± 4.5	1.34 ± 0.06	0.35 ± 0.05	13.61 ± 0.26	96.7 ± 2.6	74.5 ± 2.2

Strains were cultivated in 50 mL of a low carbonate, Na^+^-depleted complex phosphate medium (PY-Na^+^) for 13 days at 30 °C, with the headspace pressurized to 1 bar overpressure with N_2_. Values are average ± standard deviations from biological triplicates. Yield coefficients (Y) are expressed as mol mol^−1^ (product) or g mol^−1^ (biomass)

^*^ CO_2_ production was estimated based on stoichiometry

Under low carbonate and Na^+^-depleted conditions, the five *A. woodii* strains exhibited distinct metabolic profiles during fructose fermentation. AWO_CDM∆*lysR*:p_RS05465_ was the only strain which completely consumed the fructose supplemented (64.6 ± 2.8 mM), whereas for AWO_WT, AWO_CDM, AWO_CDM∆*lysR*:p_MeOH_1_ and AWO_CDM∆*lysR* fructose consumption was 85, 72, 86, 71%, respectively. Biomass formation was comparable for AWO_CDM, AWO_CDM∆*lysR* and AWO_CDM∆*lysR*:p_MeOH_1_ (Y_X/Fru_ = 13.4 to 13.7 g mol^−1^) but was significantly lower for AWO_WT and AWO_CDM∆*lysR*:p_RS05465,_ for which the biomass yield was only 38 and 54%, respectively, compared to the other strains. Metabolite production revealed distinct strain-specific profiles. For AWO_WT, acetate production dominated (90.9 ± 2.4 mM), yielding 1.71 ± 0.07 mol_Ace_mol_Fru_^−1^. Additionally, small amounts of ethanol were produced (Y_EtOH/Fru_ = 0.09 ± 0.01 mol mol^−1^). In contrast, AWO_CDM∆*lysR*:p_RS05465_ reached the highest ethanol yield (0.87 ± 0.01 mol mol^−1^), which is 9.7, 3.3, 3.8 and 2.5 times higher than that of AWO_WT, AWO_CDM, AWO_CDM∆*lysR* and AWO_CDM∆*lysR*:p_MeOH_1_, respectively. For AWO_CDM∆*lysR*:p_RS05465_, a reversed metabolic profile was observed in which more ethanol (56.5 ± 2.1 mM) than acetate (28.2 ± 1.7 mM) was produced, corresponding to an EtOH:Ace ratio of 2. Interestingly, AWO_CDM∆*lysR*:p_MeOH_1_ expressing *adhE* from the p_MeOH_1_ promoter also exhibited significant ethanol production (0.35 ± 0.05 mol mol^−1^), which is slightly higher than the yield for AWO_CDM in which *adhE* is not overexpressed. This result was unexpected, as testing of p_MeOH_1_ with pFAST showed it to be strictly methanol-inducible (Fig. 4). This observation suggests that one of the other, endogenous alcohol dehydrogenases of *A. woodii* (e.g., Adh4 and Adh6) might be upregulated under Na^+^-depleted conditions and thereby contribute to ethanol formation, as previously demonstrated with an *∆adh4 A. woodii* mutant [49]. Additionally, pyruvate and lactate were minor products for all strains (≤ 3.3 mM). Balancing of carbon and electrons showed relatively high recovery for all strains, with the notable exception of AWO_CDM∆*lysR*:p_RS05465_ which initially showed lower recovery of 73.1 ± 2.4% and 64.9 ± 1.7% for carbon and electrons, respectively. This deficit suggested carbon flux diverted toward unquantified products. Subsequent amino acid analysis identified alanine (7.0 ± 0.2 mM) and valine (4.8 ± 0.8 mM) as additional products of AWO_CDM∆*lysR*:p_RS05465_ under these conditions and taking these products into account resulted in an increased carbon and electron recovery of 86.5 ± 2.9% and 80.8 ± 2.3%, respectively. Nevertheless, the balances remain rather low, suggesting that a carbon and electron-containing compound remains unquantified.

### Ethanol production from C1 substrates

Following successful implementation of ethanol production in *A. woodii*, we next tested the two engineered strains AWO_CDM∆*lysR*:p_RS05465_ and AWO_CDM∆*lysR*:p_MeOH_1_ for ethanol production from liquid (methanol and formate) and gaseous (formate + syngas) C1 substrates (Table 2).

**Table 2:** Ethanol production from formate + syngas, formate + CO, and methanol + CO_2_ in engineered *A. woodii* strains

Strains	Substrates in [mM] or bar			Biomass (X) [g L^−1^]	Products [mM]			Yields			Balance [%]	
	Composition	Supplied	Consumed		Acetate	Ethanol	CO_2_*	Y_Ace/S_ [mol mol^−1^]	Y_EtOH/S_ [mol mol^−1^]	Y_X/S_ [g mol^−1^]	Carbon	Electron
AWO_CDM	MeOH	74.0 ± 3.9	69.7 ± 2.1	0.30 ± 0.001	47.9 ± 2.8	0.0 ± 0.0	63.9 ± 3.7	0.69 ± 0.05	0.0 ± 0.0	4.2 ± 0.1	104.4 ± 4.7	102.1 ± 7.1
	N_2_/CO_2_ (80/20)	1.0	0.1									
AWO_CDM∆l*ysR*:p_RS05465_	MeOH	61.3 ± 0.7	56.5 ± 2.4	0.30 ± 0.02	37.3 ± 0.7	5.4 ± 0.6	24.9 ± 0.4	0.66 ± 0.04	0.1 ± 0.01	5.3 ± 0.59	118.0 ± 6.7	120.4 ± 9.4
	N_2_/CO_2_ (80/20)	1.0	0.8									
AWO_CDM∆*lysR*:p_MeOH_1_	MeOH	62.1 ± 2	59.6 ± 1	0.20 ± 0.07	39.6 ± 0.1	2.1 ± 0.6	26.4 ± 0.1	0.61 ± 0.08	0.03 ± 0.01	4.1 ± 0.36	106.1 ± 1.3	104.3 ± 1.7
	N_2_/CO_2_ (80/20)	1.0	0.2									
AWO_CDM	Formate	140.5 ± 5.6	139.1 ± 4.4	0.07 ± 0.01	34.3 ± 1.2	0.0 ± 0.0	69.5 ± 2.2	0.25 ± 0.01	0.0 ± 0.0	0.47 ± 0.03	100.2 ± 1.9	100.5 ± 3.9
	N_2_/CO_2_ (80/20)	1.0	0.2									
AWO_CDM∆*lysR*:p_RS05465_	Formate	167.2 ± 2.1	167.2 ± 2.1	0.07 ± 0.02	42.4 ± 0.6	0.0 ± 0.0	83.6 ± 1.0	0.25 ± 0.01	0.0 ± 0.0	0.43 ± 0.1	102.2 ± 0.3	104.5 ± 0.6
	N_2_/CO_2_ (80/20)	1.0	0.1									
AWO_CDM^***^	Formate	180.0 ± 0.1	91.6 ± 6.6	0.05 ± 0.04	30.3 ± 0.8	0.0 ± 0.0	45.8 ± 3.3	0.33 ± 0.02	0.0 ± 0.0	0.88 ± 0.01	119.5 ± 3.1	139.3 ± 6.1
	CO (100)	1.0	0.0									
AWO_CDM∆*lysR*:p_RS05465_	Formate	198.06 ± 13.3	134.1 ± 13.7	0.06 ± 0.01	30.8 ± 1.9	1.4 ± 0.2	67.0 ± 6.8	0.23 ± 0.02	0.01 ± 0.0	0.48 ± 0.07	103.4 ± 2.6	109.0 ± 5.2
	CO (100)	1.0	0.0									
AWO_CDM	Formate	123.1 ± 1.4	123.1 ± 1.4	0.34 ± 0.05	136.9 ± 6.8	1.4 ± 0.2	**	**	**	**	**	**
	0.34 ± 0.05	136.9 ± 6.8	1.4 ± 0.2									
AWO_CDM∆*lysR*:p_RS05465_	Formate	130.8 ± 9.7	130.8 ± 9.7	0.28 ± 0.04	128.0 ± 6.5	11.3 ± 1.4	**	**	**	**	**	**
	Syngas CO_2_/H_2_/CO (10/60/30)	1.3	0.6									

Strains were cultivated in 50 mL of a complex phosphate medium (PY) for 13 days at 30 °C, with the headspace pressurized to 1 bar overpressure with N_2_/CO_2_ (80/20%), CO (100%) or Syngas CO_2_/H_2_/CO (10/60/30%). Values are average ± standard deviations from biological triplicates. n.a., substrate not consumed. Yield coefficients (Y) are expressed as mol mol^−1^ (product) or g mol^−1^ (biomass)

^*^ Total CO_2_ production was estimated based on the theoretical stoichiometry of methanol and formate fermentation. Negative values indicate CO_2_ consumption

^**^ Gas consumption was not quantified. Therefore, no estimation of CO_2_ production and yield calculation was possible

^***^ Data shown are from a duplicate experiment

The metabolic product ethanol is highly reduced (degree of reduction ᵹ = 6) and can therefore only be generated with high carbon efficiency if the carbon source used for production is highly reduced [59]. As such, methanol was selected as one promising substrate because it is a highly reduced carbon source with rather favorable bioenergetics which might be able to favor formation of ethanol in addition to acetate. On methanol + CO_2_, the reference strain AWO_CDM did not produce ethanol, whereas the engineered strains redirected part of the carbon flux toward ethanol, with AWO_CDM∆*lysR*:p_RS05465_ producing 5.4 ± 0.6 mM ethanol (Y_EtOH/S_: 0.10 ± 0.01 mol mol^−1^) and AWO_CDM∆*lysR*:p_MeOH_1_ reaching 2.1 ± 0.6 mM (Y_EtOH/S_: 0.03 ± 0.01 mol mol^−1^). Biomass concentrations of the engineered strains (0.2–0.3 g L^−1^) were slightly lower than AWO_CDM (0.3 g L^−1^) and acetate yields decreased marginally but insignificantly from 0.69 ± 0.05 mol mol^−1^ in AWO_CDM to 0.66 ± 0.04 and 0.61 ± 0.08 mol mol^−1^ in the engineered strains AWO_CDM∆*lysR*:p_RS05465_ and AWO_CDM∆*lysR*:p_MeOH_1_, respectively, indicating a partial diversion of carbon from acetate to ethanol.

On formate, AWO_CDM and AWO_CDMΔ*lysR*:p_RS05465_ reached similar biomass concentrations of ~0.07 g L^−1^ and both strains stoichiometrically converted formate to acetate (Y_Ace/S_ ~ 0.25 mol mol^−1^) without detectable ethanol production. In contrast to methanol, formate is a rather oxidized substrate with limited bioenergetics. Therefore, we also tested whether the combination of formate and syngas could enable ethanol production compared to formate alone. On formate + syngas, both strains used all available formate along with part of the syngas. Compared to growth on formate alone, biomass concentrations were increased 4.4 and 3.6-fold for AWO_CDM and AWO_CDMΔ*lysR*:p_RS05465_, respectively. Ethanol production was observed for both strains, but the final concentration was 8.1-fold higher for AWO_CDMΔ*lysR*:p_RS05465_ compared to AWO_CDM, with comparable acetate production by both strains.

Collectively, these results successfully demonstrate ethanol production from methanol and show increased production of ethanol from formate + syngas in *A. woodii.* AWO_CDMΔ*lysR*:p_RS05465_ showed higher ethanol formation compared to AWO_CDMΔ*lysR*:p_MeOH_1_ and the parental strain AWO_CDM, which produced ethanol from formate + syngas but not from formate or methanol + CO_2_.

## Discussion

In this work, we characterized a suite of eight constitutive (six native to *A. woodii* and two exogenous) and a methanol-inducible (p_MeOH_1_) promoter using the pFAST reporter system. Then, we used the strongest characterized promoter (p_RS0465_) and p_MeOH_1_ to replace the native promoter of the bifunctional aldehyde/alcohol dehydrogenase AdhE of *A. woodii* using the endogenous CRISPR type I-B system. Finally, we conducted small-scale batch fermentations of the engineered strains and demonstrated ethanol production from fructose, methanol and formate + syngas.

Employing characterized promoters is a critical step in the rational engineering of microbial strains, as it enables precise control over gene expression for pathway optimization. While commonly used inducible systems offer temporal control, they also carry inherent limitations, such as the need for antibiotics (e.g., aTc) or metabolizable inducers which might interfere with cell physiology and/or affect process economics. In contrast, the native constitutive promoters characterized in this study provide tunable expression without exogenous inducers. Additionally, the native methanol-inducible promoter provides an inducible system based on a carbon and energy source which is highly desirable for bioproduction. Among the eight promoters characterized, three promoters linked to the WLP surprisingly showed either little (p_AcsD_) or no fluorescence (p_CooS_ and p_AcsE_) when tested with pFAST, although the expression of the genes involved in this central pathway is strong and constitutive. Indeed, transcriptomic data [52] revealed that the genes downstream of p_AcsD_, p_CooS_ and p_AcsE_ are expressed at high levels across a broad range of substrates (e.g., ~ 4000, 2000 and 9000 TPM on fructose, respectively). The gene controlled by the strongest promoter we evaluated (p_RS05465_) displayed a robust yet generally lower expression profile across conditions (e.g., ~ 1500 TPM on fructose). Taken together, the discrepancy we observed between the RNA-seq expression values and our reporter assays underline the necessity of characterizing promoters experimentally, especially in non-model hosts like *A. woodii*.

This apparent disparity between transcriptomic data and our promoter characterization assays can be attributed to several factors. First, TPM values reflect not only transcriptional initiation but also mRNA stability; dynamic differences in transcript decay rates can substantially uncouple measured expression from the underlying promoter strength [14, 16, 38, 43, 53]. Second, RNA-seq data only provide insights into the transcriptional landscape, whereas reporter assays incorporate effects of translational regulation that may strongly influence promoter activity. Third, the selected sequences may not fully represent native promoter structures, as selection was based on the σ⁷⁰ consensus of *Clostridia* [33]. Some target genes could instead be controlled by alternative σ factors or transcriptional regulators, which could have an important impact if the chosen promoters were too short to include relevant binding sites.

Inducible promoters are attractive tools for controlled expression of larger transcription units while mitigating metabolic burden [21, 57]. In addition to constitutive promoters, a methanol-inducible promoter (p_MeOH_1_) was therefore also characterized in *A. woodii* using pFAST. p_MeOH_1_ was activated by methanol in a dose-dependent manner, yet its activity was strongly influenced by other carbon sources: while ethanol permitted induction, fructose and formate caused strong repression. This reflects a clear substrate hierarchy in which methanol utilization is subordinate to preferred carbon sources and is reminiscent of carbon catabolite repression regulation reported in acetogens, where methanol utilization is suppressed in the presence of more favorable substrates such as glucose [46]. The reason for this behavior is most likely linked to the fact that the cultures were performed in batch mode with fructose or formate present in excess, a regime that favors complete repression of methanol utilization until the preferred substrate is depleted. In contrast, co-consumption of methanol and formate was observed in formate-limited chemostat cultures of *Acetobacterium* sp. [3] and co-consumption of fructose and formate or formate with gaseous feedstocks was observed in substrate-limited chemostat cultures with *A. woodii* [52]. These observations suggest that methanol catabolism and p_MeOH_1_-driven expression in *A. woodii* is dependent both on the type of co-substrate and the cultivation mode but the underlying regulatory network remains to be elucidated.

The regulatory mechanism underlying p_MeOH_1_ activity likely involves two layers of control. The methanol methyltransferase operon (*mta*) in *A. woodii* harbors a transcriptional regulator, *mtaR* (Awo_c22810), situated directly upstream of the other genes and downstream of p_MeOH_1_ [41, 45]. MtaR shares 38% identity with PocR from *Salmonella typhimurium* [41], a regulator known to activate metabolic pathways in response to small-molecule effectors [19, 63]. Because of this structural similarity, MtaR was proposed to activate the expression of the *mta* operon in the presence of methanol [41]. In addition to MtaR, carbon catabolite repression (CCR) likely accounts for the repression observed in the presence of fructose and possibly with formate as methanol was consumed only after fructose was fully depleted in co-feeding experiments and was not consumed in the presence of formate. CcpA is a global regulator of CCR in Gram-positive bacteria, repressing non-preferred substrate pathways in the presence of rapidly metabolizable sugars [28]. Importantly, the genome of *A. woodii* bears a gene encoding CcpA (Awo_c13680, sharing 83% and 45% identity with CcpA of *Acetobacterium bakii* and *C. ljungdahlii*, respectively). A putative CcpA-binding motif (cre: ATTACAATTGGTTAAT) overlapping the − 10 element of p_MeOH_1_ was identified using a *Clostridia* consensus sequence, ATGAAAACGTTTTAAT [55], suggesting direct transcriptional occlusion may occur upon CcpA binding, i.e., when cells are grown with preferred carbon sources. Together, these findings suggest that the regulation of the *mta* operon is orchestrated by a mechanism in which MtaR mediates methanol-dependent induction, while CcpA ensures repression in the presence of fructose or formate.

*A. woodii* was previously shown to naturally produce ethanol from fructose with a relatively high yield (up to 0.79 mol mol^−1^) under Na^+^ depletion, a condition under which the activity of the WLP is severely inhibited. As a result, CO_2_ cannot efficiently serve as an electron sink, which stimulates ethanol production via the NADH-consuming AdhE enzyme [49]. In this work, we showed that increasing the expression of the native *adhE* gene via a precise genome edit is sufficient to significantly increase the ethanol yield from fructose under Na^+^ depletion (0.87 mol mol^−1^), demonstrating that promoter strength alone is a key limiting factor for ethanol production in *A. woodii*. This has also been previously observed in other organisms, such as *C. acetobutylicum*, where targeted promoter replacement in the native cellulase operon was sufficient to enable growth on cellulose [75]. In our design, *lysR*, located directly upstream of *adhE*, was deleted together with the native promoter region and replaced by either p_RS05465_ or p_MeOH_1_ to relieve potential local regulation of *adhE* expression. Because LysR-type regulators can control multiple targets, we additionally constructed a control strain lacking *lysR* but retaining the native *adhE* promoter (AWO_CDMΔ*lysR*) to separate effects of *lysR* deletion from those of promoter replacement. The control strain did not display the pronounced shift toward ethanol observed in the promoter-replacement strains, suggesting that the main driver of the ethanologenic phenotype is increased *adhE* expression. However, more subtle effects of *lysR* deletion on other genes cannot be fully excluded, which will require further systems-level analyses.

The biotechnological potential of *A. woodii* relies on the WLP for the conversion of C1 feedstocks, which requires conditions under which the WLP is fully active. We therefore investigated whether the engineered strains overexpressing *adhE* could produce ethanol from several C1 substrates (methanol + CO_2_, formate, formate + CO and formate + syngas). Ethanol is a more reduced product than acetate and its synthesis therefore requires a greater input of reducing equivalents. Moreover, diverting carbon flux toward ethanol comes at the cost of reduced acetate production, which is a major driver of ATP generation via the WLP. Consequently, the feasibility of ethanol production from C1 substrates is expected to depend on two key factors: the ATP yield supported by the substrate and the availability of reducing equivalents. Substrates that satisfy both criteria such as methanol or syngas, which provide high amounts of ATP and flexible routing of reducing equivalents [42, 45] are therefore the most promising candidates.

Consistent with this hypothesis, no ethanol production was detected on formate alone, in agreement with the negative ATP yield predicted by the stoichiometric model (−0.09 ATP mol^−1^ of ethanol, [59]). In contrast, both methanol and formate + syngas supported ethanol production with a selectivity of 12.6 and 8.1%, respectively, confirming that these substrates provide sufficient bioenergetics to sustain carbon flux toward ethanol, in line with model predictions (0.70 and 0.08 ATP mol^−1^ of ethanol, respectively, [52, 59]). However, despite favorable bioenergetics (0.36–1.27 ATP mol^−1^ of ethanol, depending on the formate:CO stoichiometry), only trace amounts of ethanol were produced from formate + CO, which might be attributed to the well-documented inhibitory effect of CO on growth [8, 32, 72]: the resulting low growth rates possibly limit acetyl-CoA availability, reducing the carbon flux that could otherwise be directed toward ethanol synthesis.

Together with our promoter replacement strategy, these findings underline that ethanol formation in *A. woodii* is constrained both by energy conservation and by endogenous transcriptional control. Indeed, relieving energetic bottlenecks through the selection of energetically favorable substrates and decoupling *adhE* expression from its native promoter suffice to enable the production of ethanol from C1 feedstocks, illustrating how small, targeted genome edits can significantly reshape carbon flux and unlock latent metabolic traits.

## Conclusion

This work establishes a modular plasmid assembly platform and a set of characterized promoters for *A. woodii*, enabling rapid and standardized control of gene expression and CRISPR-based genome editing. Applying this toolbox by implementing a single promoter swap at the *adhE* locus, we redirected carbon flux from acetate toward ethanol and demonstrated ethanol production from methanol + CO_2_ and formate + syngas in *A. woodii*. Together with its robustness and its ability to convert diverse C1 substrates, these tools position *A. woodii* as a genetically accessible chassis for sustainable production of chemicals and fuels from CO_2_-derived and renewable C1 feedstocks.

## Supplementary Information

Supplementary Material 1.

## Data Availability

The data generated and analyzed in this study are included in the main manuscript or supplementary material. Raw datasets for this study can be accessed through the TU Wien Research Data Repository (https://doi.org/10.48436/wmzp4-c1597).
